# Realization of an ultrathin acoustic lens for subwavelength focusing in the megasonic range

**DOI:** 10.1038/s41598-018-27312-5

**Published:** 2018-06-14

**Authors:** Jaeyub Hyun, Yong Tae Kim, Il Doh, Bongyoung Ahn, Kyungmin Baik, Se-Hwa Kim

**Affiliations:** 1 Center for Medical Convergence Metrology, Korea Research Institute of Standards and Science (KRISS), 267 Gajeong-ro, Yuseong-gu, Daejeon, 34113 Republic of Korea; 2 Center for Nano-Bio Measurement, Korea Research Institute of Standards and Science (KRISS), 267 Gajeong-ro, Yuseong-gu, Daejeon, 34113 Republic of Korea; 3 Department of Medical Physics, Korea University of Science and Technology, 176 Gajeong-ro, Yuseong-gu, Daejeon, 34113 Republic of Korea

## Abstract

In this study, we report the first experimental realization of an ultrathin (0.14λ, λ = 1.482 mm means wavelength at 1 MHz in the water medium) subwavelength focusing acoustic lens that can surpass the Rayleigh diffraction limit (0.61λ/NA, NA means numerical aperture). It is termed a Super-Oscillatory Acoustic Lens (SOAL), and it operates in the megasonic range. The SOAL represents an interesting feature allowing the achievement of subwavelength focusing without the need to operate in close proximity to the object to be imaged. The optimal layout of the SOAL is obtained by utilizing a systematic design approach, referred to here as topology optimization. To this end, the optimization formulation is newly defined. The optimized SOAL is fabricated using a photo-etching process and its subwavelength focusing performance is verified experimentally via an acoustic intensity measurement system. From these measurements, we found that the proposed optimized SOAL can achieve superior focusing features with a Full Width at Half Maximum (FWHM) of ~0.40λ/NA ≃ 0.84 mm (for our SOAL, NA = 0.707) with the transmission efficiency of 26.5%.

## Introduction

The achievement of the subwavelength focusing feature, which focuses acoustic power into a very small region, is a most important task in the medical ultrasound field, from diagnostics^1^ to therapeutics^2^. The achievement of subwavelength focusing makes it possible to discover and cure very tiny substances, such as cancer and tumors, which not easily detected by typical medical ultrasound imaging and therapy systems. In other words, such an achievement indirectly implies that one can provide better medical service to more people. Unfortunately, current systems have a focusing limit which relates approximately to the wavelength of the wave used. Formally, the focusing limit, *d*, is limited by the wavelength of the wave used, λ, and the Numerical Aperture (NA) of the imaging system as *d* = 0.61λ/NA (i.e., the *Rayleigh diffraction limit*)^3,4^. Meanwhile, to overcome this diffraction limit and realize acoustic imaging and therapy systems with subwavelength focusing performance, acoustic lenses can be considered as a viable solution. In general, acoustic lenses can be classified into two types according to the utilization of evanescent waves. Evanescent waves are an important factor in terms of focusing and imaging, because these types of waves contain subwavelength fine information pertaining to the object^5^.

More specifically, acoustic lenses which utilize an evanescent wave can be realized through an artificially designed microstructure known as an Acoustic MetaMaterial (AMM). Examples include acoustic superlenses^6^ and acoustic hyperlenses^7^. AMM-based lenses demonstrate extremely improved focusing limits (0.05λ/NA in^8^). However, the object to be imaged should be placed close to the lens for the coupling of the near-field evanescent wave to the lens. In other words, it is difficult to apply these AMM-based lenses to conventional far-field imaging methods based on a pulse-echo scheme in which the object would be placed far away from the lens^9–11^. Moreover, although the principle of the AMM-based lenses has been experimentally proven^6–8,12^, manufacturing a microstructure of such a lens in the megasonic range (>1 MHz) involves significant challenges related to fabrication quality and the control of energy losses. Hence, if one can realize an acoustic lens with a subwavelength focusing feature without using an evanescent wave, it will be a very powerful solution for clinical applications in practice. Based on this need, a conventional refractive-type concave acoustic lens which is attached to the front of the source transducer is commonly used^13^. This type of acoustic lens has the advantages of easy fabrication and far-field imaging. However, its range of application is limited generally due to both its large size and poor detachability. It is also difficult to overcome the aforementioned diffraction limit through this type of acoustic lens. Thus, to realize a more compact acoustic lens, a planar acoustic lens that can focus acoustic power by modulating the phase delay of the acoustic wave, such as a Fresnel Zone Plate (FZP) lens, was proposed^14–16^. Many previous studies on both the design and experimental realization of a planar acoustic lens are reviewed and summarized in this study (see Table S1 in the Supplementary Note). In fact, as shown in Table S1, a variety of attempts at the practical realization of a planar acoustic lens have been reported thus far, but subwavelength focusing with these planar lenses remains a difficult problem. To overcome this limitation, several Acoustic MetaSurface (AMS)-based lenses have recently been proposed^17–19^. However, because the configuration of several AMS-based lenses is highly complex (e.g., a coiled structure or a labyrinthine structure), they have been experimentally realized only for use in an air medium in the audible frequency range. For a water medium in the megasonic range, there are no practically realizable acoustic lenses due to the difficulty in manufacturing such lenses. The previous studies in this area provide evidence that an ultrathin planar acoustic lens capable of subwavelength focusing in megasonic range remains unrealized. In particular, we also focus on the megasonic range (>1 MHz) here as the target operational frequency because it is easy to measure a megasonic beam through a hydrophone with a very small aperture because the wavelength is relatively large in water. If the megasonic beam is measured through a hydrophone with an aperture of a size comparable to the wavelength, an accurate FWHM cannot be measured due to the size effect^20^.

Meanwhile, in 1952, Toraldo di Francia suggested that concepts of super-directive antennas could be applied to optical instruments to increase their spatial resolution beyond the diffraction limit^21^. The super-directive antenna could make an arbitrarily narrow beam by precisely tailoring the interference of waves. Since then, the idea of super-directive antennas has been extended to the phenomenon as now known today as super-oscillation through many studies^5,22–26^. In 2012, in the optical field, one study revealed that a remarkable planar optical lens known as a Super-Oscillatory Lens (SOL) is capable of subwavelength focusing^27^. The concept of super-oscillation used in that study^27^ corresponds to a phenomenon in which an image waveform oscillates even more rapidly than the highest constitutive frequency component of the original image waveform in the main focusing area. That study achieved the subwavelength focusing feature at a position 10λ away from the optical lens, with the configuration designed using a Particle Swarm Optimization (PSO) algorithm. This was the first report of the concept of an SOL being applied to optical imaging as a practical solution for subwavelength focusing and imaging. The attractive feature of the SOL is that it can provide subwavelength focusing without utilizing evanescent waves. In other words, the subwavelength focusing feature can only be achieved through a propagation wave. Therefore, unlike existing metamaterial-based lenses, there is no need to place an object very close to the SOL. This implies that it is possible to realize far-field subwavelength focusing through the SOL. In the acoustic field, as a counterpart to this optical SOL, the feasibility of realizing the acoustical super-oscillation phenomenon was examined in 2014^28^. That study theoretically achieved subwavelength focusing by adjusting the radius of a piezoelectric annular ring. Unfortunately experimental realization was not carried out. Moreover, the ideal assumption of no mechanical crosstalk between neighboring annular ring-type piezoelectric elements was considered. Meanwhile, practical design factors including the source condition, operational frequency, target subwavelength focusing area, and lens thickness should be considered during the design process in order to obtain a realizable and applicable SOAL. The aforementioned studies used design methodologies based on a heuristic algorithm, such as PSO or a Genetic Algorithm (GA)^27,29^, to consider these design factors. These design methodologies have the advantage of easy implementation, but the computational cost is very high and the process is time consuming. Also, layouts of the designed SOAL are limited to simple configurations.

Thus, this study describes the development of a systematic inverse design process of a SOAL based on topology optimization to determine the optimal material distribution in a desired design domain^30^ so as to resolve several disadvantages of existing design methodologies and achieve an efficient design. Furthermore, to the best of our knowledge, this is the first study in which an optimized SOAL is experimentally realized and the subwavelength focusing feature is achieved in the megasonic range.

## Results

### Demonstration of the super-oscillation phenomenon and its extension to a subwavelength focusing acoustic lens

Above all, the super-oscillation mechanism of the proposed megasonic SOAL can be demonstrated simply using a one-dimensional (1D) wave composed of six spatial Fourier components, following a previous earlier approach^31^. The 1D super-oscillating function can be defined by *f*(*r*) in Eq. (1), where *r* is the lateral position normalized by the radius of a source transducer.

1 $$f(r)={\sum }_{n=0}^{n=5}{A}_{n}{e}^{j2\pi nr}$$

**Figure 1:**
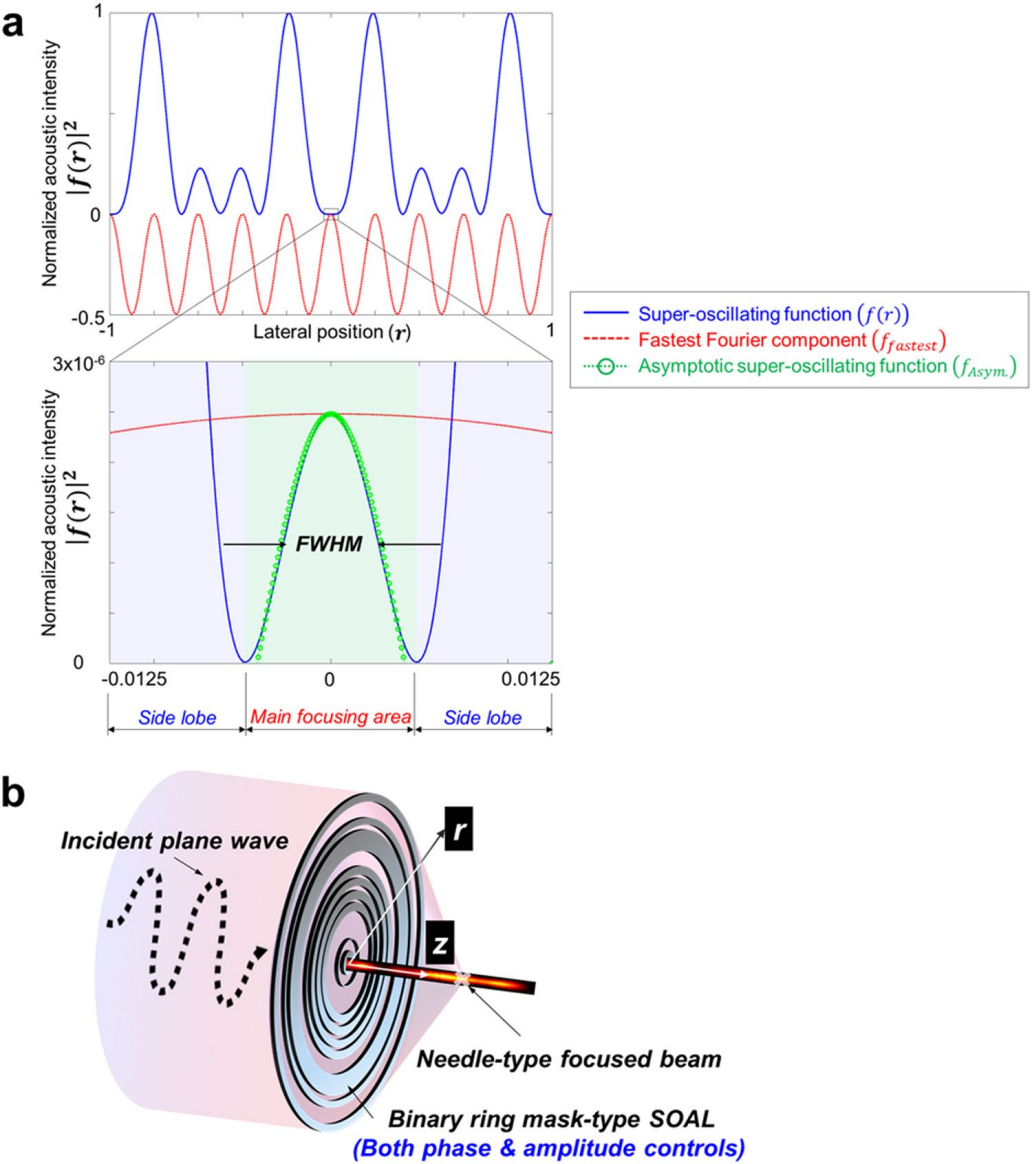
Megasonic SOAL and its fundamental mechanism. (**a**) Fundamental mechanism of the 1D super-oscillating function. Upper panel: the super-oscillating function (Eq. (1), solid blue line) and corresponding fastest Fourier component $\,{f}_{fastest}=P\,\cos (10{\rm{\pi }}r)$ (dashed red line). Lower panel: zoomed-in view of the function near *r* = 0. There is a narrow peak, about ten times narrower than the fastest Fourier component of the original wave near *r* = 0. This narrow peak can be approximated by ${f}_{Asym}=P\,\cos (500{\rm{\pi }}r)$ (dotted-circle green line). The super-oscillating function can be separated into the two regions: the desired main focusing area and the undesired side lobe. **(b)** A SOAL with a binary ring mask controls both the phase and amplitude. Through the SOAL, the incident original acoustic plane wave can be transformed into a needle-type focused beam.

In this equation, *A*_*n*_ denotes the Fourier coefficients (here, we use *A*_0_ = 19.0123, *A*_1_ = −2.7348, *A*_2_ = −15.7629, *A*_3_ = −17.9047, *A*_4_ = −1.0000, *A*_5_ = 18.4910). In Fig. 1a, we show the acoustic intensity of the original wave ${|f(r)|}^{2}$ (i.e., solid blue line) and its fastest Fourier component ${f}_{fastest}=P\,\cos (10{\rm{\pi }}r)$ (i.e., dashed red line). Here, the term “fastest” refers to the spatially fastest oscillating component by the components of the original wave at the highest frequency. As represented in the lower panel in Fig. 1a, we note that there is a narrow peak, about ten times narrower ${f}_{Asym}=P\,\cos (500{\rm{\pi }}r)$ than the fastest Fourier component of the original wave near *r* = 0. From this 1D demonstration, we can find that super-oscillation refers to a waveform which oscillates faster than the highest constitutive frequency components of the original wave, in the finite interval (e.g., the desired main focusing area). Thus, it is important to note that the subwavelength focusing feature is guaranteed only in this finite area. As shown in this simple 1D demonstration, subwavelength focusing based on the super-oscillation phenomenon can be achieved by controlling both the amplitude (*Fourier coefficient*) and the phase (2*πnr*) of the wave diffracted from the micro-slit of the lens.

Meanwhile, the subwavelength feature of a super-oscillatory wave should be accompanied by high-amplitude regions outside the desired main focusing area. This undesired region is termed a side lobe. In order to improve the focusing performance of the lens (i.e., an even narrower FWHM), an undesired side lobe is an inevitable result because the relationship between the desired and undesired regions has trade-off. Indeed, the SOAL can achieve any amount of FWHM or resolution at the expense of side lobes. Therefore, it is necessary to select the proper size of the desired main focusing area when attempting to design the SOAL practically, in order to make the side lobe smaller than the main focusing area. As shown in Fig. 1b, the SOAL can be realized through a binary ring mask with a spatially varying phase and amplitude. The binary ring mask-type SOAL is a promising technology for achieving subwavelength focusing based on the super-oscillation phenomenon^27^. It can be manufactured easily using existing microfabrication techniques, such as a photo-etching process^32^. Given this advantage, we selected the binary ring mask-type SOAL as a means to realize subwavelength focusing in this study. The aforementioned original wave can be transformed into a super-oscillatory waveform with the subwavelength focusing feature using this SOAL. In the next section, we present the physically quantifiable optimization formulation used to design the optimal layout of the binary ring mask-type SOAL.

### The optimal layout of the megasonic super-oscillatory acoustic lens

**Figure 2:**
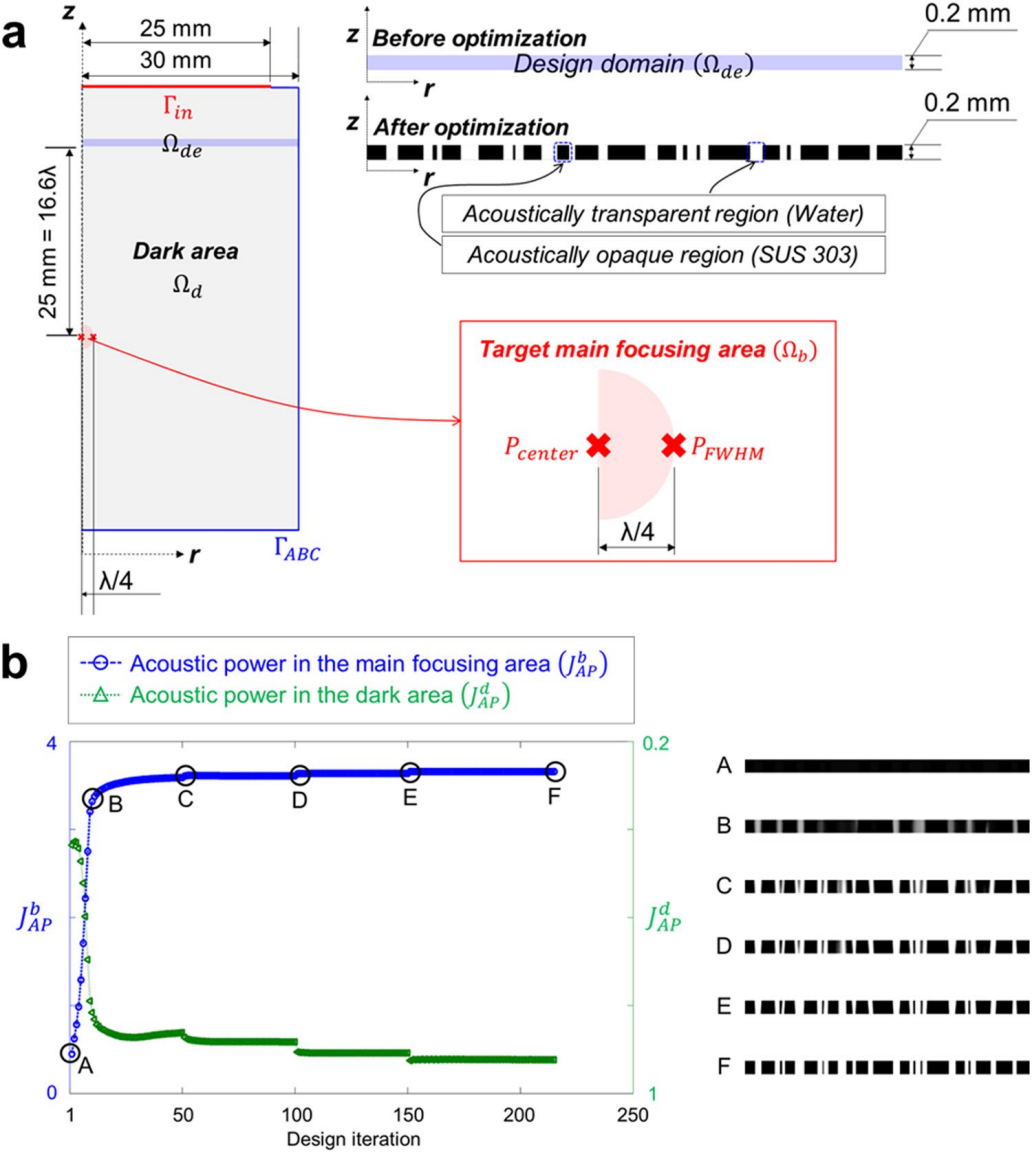
Numerical model for both the analysis and design of the binary ring mask-type SOAL. **(a)** Configuration of the numerical model for the topological design of the layout of the binary ring mask-type SOAL. The red crosses in the red box indicate the specific points to compute the constraint (*g*_2_) related to the target FWHM. **(b)** Topology optimization history. The image shows the evolution of the 2D topological layout of the SOAL and the acoustic power during the optimization process. The black area represents the SUS 303 material, while the white region represents water. The acoustic power $({J}_{AP}^{b})$ in the main focusing area gradually increases. The points from A to F in the figure indicate the design iterations selected to show the evolution of the optimal layout. From the optimal layouts of SOAL obtained in these selected design iterations (Right panel in b), we confirm that layout of the SOAL converges well during the optimization process. The initial layout (i.e., point A) shows a nearly black-scale rectangle because the optimization process starts with the area fully filled with the solid material (i.e., SUS 303). The final layout (i.e., point F) of the SOAL converges well.

We used topology optimization, which is one of the most flexible types of inverse design methods, since topological changes such as increase or decrease in the number of holes in the desired design domain are allowed during the design optimization process^30^. To obtain the optimal layout of the binary ring mask-type SOAL, a numerical model should be considered in both the analysis and design steps. Figure 2a shows the configuration of the numerical model. Here, we used the two-dimensional (2D) axisymmetric finite element model to solve the acoustic wave propagation problem in the megasonic range efficiently based on the Helmholtz equation. The region for the optimal layout of the SOAL (i.e., the design domain, Ω_*de*_ in Fig. 2a) is located at a position 10 mm away from the source transducer (Γ_*in*_ in Fig. 2a), which can be modeled using a plane wave boundary condition (Γ_*ABC*_ in Fig. 2a). We enclosed the analysis and design domain with an absorbing boundary to prevent the wave reflection from the exterior boundary^33^. The radius and height (i.e., the thickness of the SOAL) of the design domain are 30 mm and 0.2 mm, respectively. The position of the target main focusing area (Ω_*b*_ in Fig. 2a) is located 25 mm (i.e., about 16.6λ) away from the design domain. Meanwhile, acoustically opaque and transparent regions are required to design the layout of the SOAL. To create these regions, in this study SUS 303 material (mass density, *ρ*_*S*_ = 8,000 kg/m^3^ and sound speed, *c*_*S*_ = 4,484 m/s) and water (*ρ*_*w*_ = 1,000 kg/m^3^ and *c*_*w*_ = 1,482 m/s) are used for the acoustically opaque and transparent regions, respectively. In this study, the shear deformation effect of elastic solid (i.e., SUS 303) was ignored during numerical simulation and optimization process of the SOAL. The Supplementary Note contains more detailed information of this issue.

As mentioned in the previous section, in principle the SOAL allows any value of FWHM. However, the larger side lobe should be accompanied in order to achieve the narrower FWHM. In particular, if the FWHM of main focusing area exceeds a certain critical value, the acoustic intensity in the side lobe becomes larger than that in the main focusing area. In other words, the increase in acoustic intensity in the side lobe should be sacrificed to design the SOAL exhibiting the even narrower FWHM. This large side lobe effect is inevitable in designing the super-oscillatory optical/acoustic lenses with very narrow FWHM^31^. Therefore, we set the target FWHM of the SOAL to ~λ/2 (red box in Fig. 2a), in order to make the acoustic intensity in the side lobe smaller than that in the main focusing area. Meanwhile, despite the possibility of designing the SOAL with the narrower FWHM, this study does not focus on the design of SOAL with FWHM narrower than λ/2 from the following three perspectives: (1) imaging (diagnosis), (2) therapeutic, and (3) thermal-viscous loss. For a detailed description of this issue, see the Supplementary Note. For a systematic design of the binary ring mask-type SOAL for subwavelength focusing, a constraint related to the target FWHM is introduced in the topology optimization setup. Generally, an acoustic lens is optimized by maximizing only the acoustic power $({J}_{AP}^{b})$ in the main focusing area^34^. However, in order to guarantee the subwavelength focusing feature of the SOAL here, we introduce a constraint (*g*_2_) related to the target FWHM in addition to an objective function $({J}_{AP}^{b})$. Note that this is a simple but very powerful optimization formulation for the design of the SOAL. In this study, the topology optimization setup used to design the layout of the SOAL is expressed as Eq. (2). In this equation, the mathematical meaning of ${\max }_{{\gamma }_{e}}{J}_{0}$ is to determine the design variables (*γ*_*e*_) that maximize the objective function expressed as *J*_0_. Here, *J*_0_ is the acoustic power in the main focusing area $({J}_{AP}^{b})$. Acoustic power can be defined by the surface integral of acoustic intensity. Also, it is worth noting that acoustic intensity is proportional to the square of acoustic pressure under the assumption of the progressive plane wave. Thus, in this study, acoustic intensity is calculated from the acoustic pressure parameters using ${|p|}^{2}/2{\rho }_{w}{c}_{w}$, with *p* being the acoustic pressure, *ρ*_*w*_ the mass density of water, and *c*_*w*_ the sound speed of water^35^. ${|p|}^{2}$ is the squared absolute pressure.

2 $$\begin{array}{c}{{\rm{\max }}}_{{r}_{e}}{J}_{0}={J}_{AP}^{b}=\frac{1}{2{\rho }_{w}{c}_{w}}{\int }_{{{\rm{\Omega }}}_{b}}{|p|}^{2}d{\rm{\Omega }}\\ {\rm{subject}}\,{\rm{to}}\,{g}_{1}=\frac{{\int }_{{{\rm{\Omega }}}_{de}}{\gamma }_{e}d{\rm{\Omega }}}{(VFF){V}_{de}}-1\le 0\\ {g}_{2}={(\frac{{{|p|}^{2}}_{{P}_{FWHM}}}{0.5{{|p|}^{2}}_{{P}_{center}}}-1)}^{2}\le \varepsilon \\ {\gamma }_{e}=[{\gamma }_{1},{\gamma }_{2},\cdots ,{\gamma }_{NE}]\in (0 \sim 1)\end{array}$$

where, *γ*_*e*_ is the design variable, which varies from 0 to 1 during the topology optimization process. *VFF* is the required volume fraction that defines the ratio of the opaque region to the entire design domain. *V*_*de*_ is the volume of the entire design domain. *ε* is the threshold value for relaxation of the constraint *g*_2_^36^. In this study, this value is set to 10^−3^. *NE* is the total number of design variables. Here, according to the updated design variable *γ*_*e*_, the material distribution in the design domain can be determined based on Eqs (3) and (4). When *γ*_*e*_ = 0, the acoustic material corresponds to water (i.e., the acoustically transparent region), and when *γ*_*e*_ = 1, the acoustic material corresponds to SUS 303 (i.e., the acoustically opaque region).

3 $$\rho ({\gamma }_{e})={(\frac{1}{{\rho }_{w}}+{{\gamma }_{e}}^{{q}_{1}}(\frac{1}{{\rho }_{S}}-\frac{1}{{\rho }_{w}}))}^{-1}$$

4 $$c({\gamma }_{e})={(\frac{1}{{c}_{w}}+{{\gamma }_{e}}^{{q}_{2}}(\frac{1}{{c}_{S}}-\frac{1}{{c}_{w}}))}^{-1}$$

In these equations, *q*_1_ and *q*_2_ are penalty factors for the mass density *ρ*, and the speed of sound *c*, respectively. These penalty factors are used to improve the convergence rate of the optimization process. These values are set to 1.5 in this study. The method of moving asymptotes (MMA) is then used as the optimization algorithm^37^. This type of optimization algorithm requires the first-order derivative information (i.e., the gradient) of the objective function, called the design sensitivity, in order to update the design variable. Thus, we carry out an efficient design sensitivity analysis based on the adjoint variable method (AVM) to compute the gradient information. Additional details about this procedure are available in the literature^38^. Figure 2b shows the evolution of both the acoustic power (i.e.,${J}_{AP}^{b}\,and\,{J}_{AP}^{d}$) and the 2D topological optimal layout of the SOAL. Here, ${J}_{AP}^{d}$ indicates the acoustic power in the dark area which is obtained by excluding the main focusing area from entire area (Fig. 2a). The initial layout shows a nearly black-scale rectangle because the optimization process starts with the area fully filled with the solid material (i.e., SUS 303). Here, in order to find pure 0–1 solutions (i.e., the perfect binary ring mask-type SOAL), a Heaviside projection filtering method with beta-continuation is applied every 50 iterations^39^. Therefore, the objective function (i.e., acoustic power ${J}_{AP}^{b}$) jumps slightly every 50 iterations, as shown in the topology optimization history plot in Fig. 2b. This optimization history confirms that the layout of the SOAL converges well during the optimization process. Moreover, the proposed systematic design methodology is equally applicable to the inverse design of a multilayer-type SOAL in addition to the aforementioned SOAL with a single layer. The Supplementary Note contains more detailed design results.

### Experimental realization of subwavelength focusing by the optimized super-oscillatory acoustic lens

To verify the subwavelength focusing performance of the optimized SOAL, we fabricated a prototype via photo-etching and conducted an experiment using an acoustic intensity measurement system to measure the acoustic field radiating from the optimized SOAL. The subwavelength focusing performance of the optimized SOAL is verified in a comparison with a conventional FZP lens with a primary focal length (i.e., the position of the target main focusing area) identical to that of the optimized SOAL. The layout of the conventional FZP lens with the specified primary focal length (*F*) can be realized using an analytical equation (Eq. (5))^40^.

5 $${b}_{n}=\sqrt{n\lambda F+{(\frac{n\lambda }{2})}^{2}}$$

**Figure 3:**
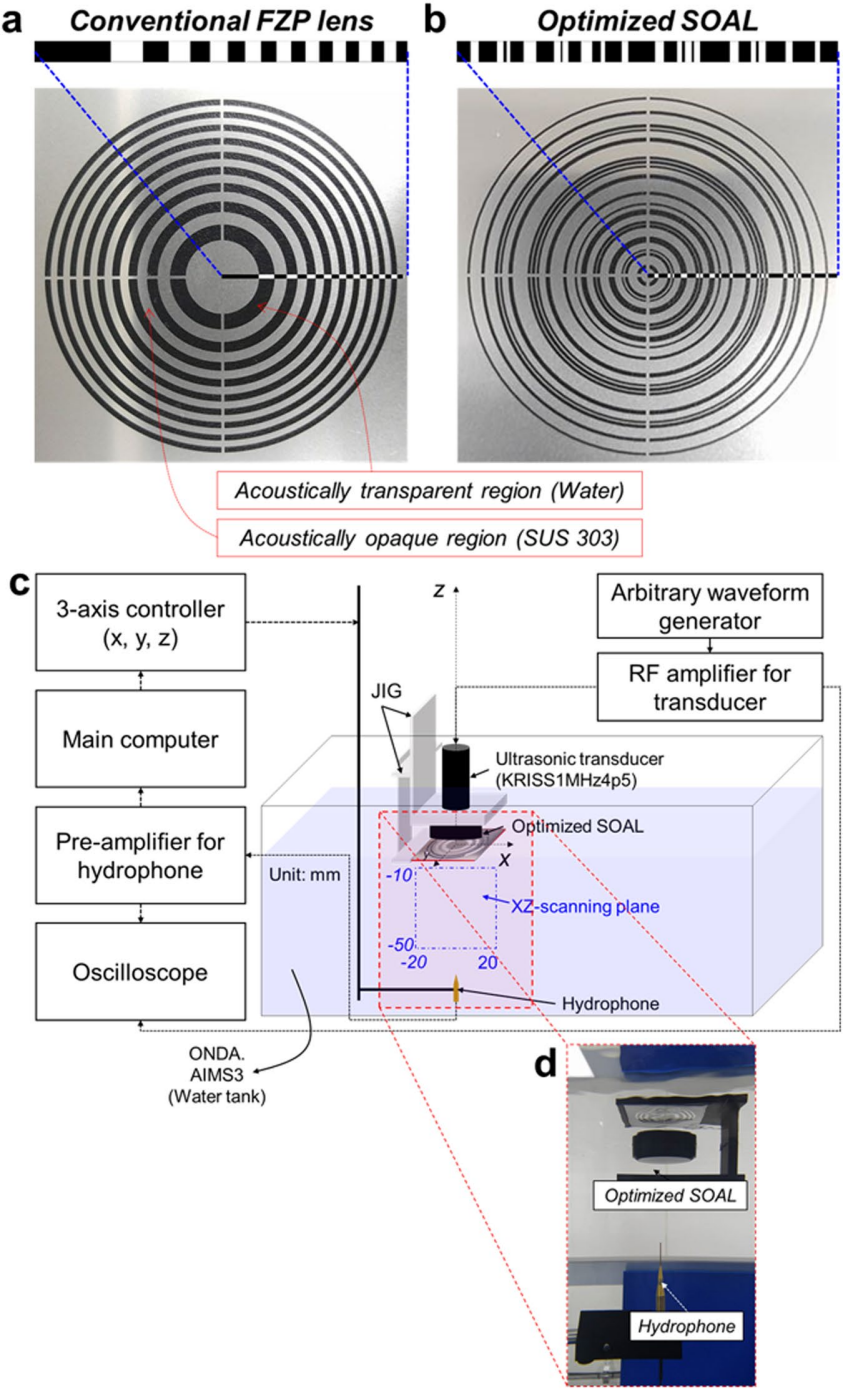
Experimental realization of the optimized SOAL. **(a)** Fabricated conventional FZP lens. The layout (i.e., dimensions of the annular zone) of the fabricated conventional FZP lens is determined by Eq. (5). (**b**) Fabricated optimized SOAL with a single layer. **(c)** Experimental setup used to measure the acoustic field through both the conventional FZP lens and the optimized SOAL. **(d)** Actual photos of both the SOAL fixed by jig and the needle-type hydrophone. Details of the dimension parameters of these fabricated lenses shown in (a,b) are presented in Table S2 in the Supplementary Note.

Here, *b*_*n*_ is the radius of the n^th^ annular zone, with *n* = 1, 2 … *N*, where is the total number of zones. *F* is the primary focal length, and *λ* is the wavelength of the acoustic wave used. For our case, the total number of zones *N* equals 19, the primary focal length *F* is 25 mm, and the wavelength *λ* is 1.482 mm (1 MHz). Both the conventional FZP acoustic lens and the optimized SOAL were fabricated using a photo-etching technique, as shown in Fig. 3a,b, respectively. Figure 3c graphically shows the experimental setup used to measure the acoustic field through the conventional FZP lens and the optimized SOAL. To measure the acoustic field effectively, we created a jig which allows the detachability of the SOAL to be adjusted easily (Fig. 3d). This jig makes it possible to test the influence of various planar lenses on the megasonic focusing performance for only one source transducer. The method section presents details of the experimental setup.

**Figure 4:**
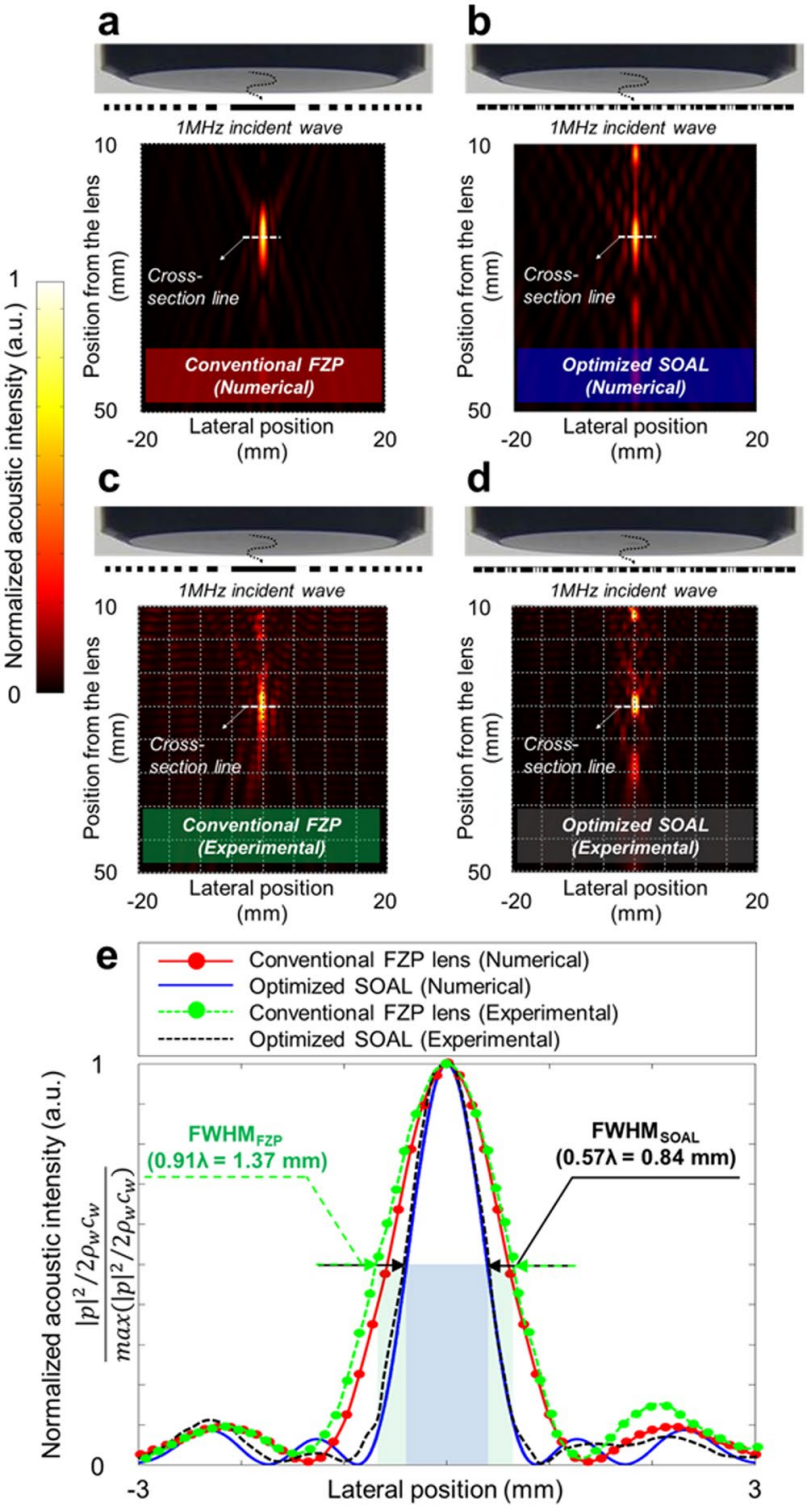
Comparison between the numerical and experimental results of the optimized SOAL with a single layer in terms of the subwavelength focusing performance. (**a**,**b**) Numerically calculated acoustic intensity fields by the conventional FZP lens (left panel, a) and the optimized SOAL (right panel, b). (**c**,**d**) Experimentally measured acoustic intensity field radiated by the conventional FZP lens (left panel, c) and the optimized SOAL (right panel, d). (**e**) Comparison of the normalized acoustic intensity fields. The cross-section line of measurement along the lateral direction is represented by the dashed white line.

Prior to showing the experimentally measured results, we present the numerically calculated results. Figure 4a,b show the numerically calculated acoustic intensity fields through the conventional FZP lens (left panel, a) and the optimized SOAL (right panel, b), respectively. Correspondingly, Fig. 4c,d show the experimentally measured acoustic intensity fields. Here, all of the results in Fig. 4 are normalized according to the maximum value of the measured acoustic intensity. The measurement is conducted on the XZ-scanning plane (i.e.,40 *mm* × 40 *mm*) in Fig. 4c. The experimental measured and the numerically calculated fields are in good agreement with each other, as shown in Fig. 4a–d. The acoustic power is concentrated very well in the target main focusing area (i.e., the primary focal length, *F* = 25 mm) through the optimized SOAL, as presented in Fig. 4b,d. Next, Fig. 4e shows a cross-sectional plot of the normalized acoustic intensity in Fig. 4a–d for a closer comparison of the FWHMs. The cross-sectional line for plotting is represented by the dashed white line. As noted above, it is essential that the NA should be taken into account for an accurate evaluation of the subwavelength focusing performance of the optimized SOAL. The NA is determined by both the radius of the input source transducer (*r*_*source*_) and the specified primary focal length (*F*), as ${\rm{NA}}=\,\sin ({\tan }^{-1}({r}_{source}/F))$. The NA in this case is approximately 0.707 for the current optimized SOAL with the single layer. Therefore, the focusing limit (i.e., the *Rayleigh diffraction limit*) of the conventional acoustic lens is 0.61λ/NA ≃ 0.86λ ≃ 1.28 mm. From the numerical and experimental results in Fig. 4 and Table 1, we find that the optimized SOAL has two significant characteristics in terms of the focusing performance. First, it has an even narrower FWHM (0.40λ/NA ≃ 0.57λ ≃ 0.84 mm) compared to that of the conventional FZP lens (0.64λ/NA ≃ 0.91λ ≃ 1.37 mm). Second, it can overcome the Rayleigh diffraction limit (0.61λ/NA ≃ 0.86λ ≃ 1.28 mm) and achieve subwavelength focusing (0.40λ/NA ≃ 0.57λ ≃ 0.84 mm). Meanwhile, the optimized SOAL has very narrow acoustic passage regions (i.e., micro-slits). In practice, these can cause thermal-viscous losses and hence a damping effect. Here, because the narrowest acoustic passage region (~0.29 mm) of the optimized SOAL is much wider than the thickness of the thermal (*δ*_*thermal*_ ~ 0.21 μm) and viscous (*δ*_*viscous*_ ~ 0.56 μm) layers estimated from a simple analytical formula^35^ at 20 °C and 1 atm, the thermal-viscous loss effect can be ignored. However, to realize a SOAL with much narrower FWHM than the current optimized SOAL, undoubtedly even narrower micro-slits should be fabricated under reasonable uncertainty. A fully coupled model of both the thermal and acoustic fields should be considered in the optimization process in future work, as the loss effect maximized by these narrower micro-slits is likely to have a significant impact on the performance of the SOAL.

**Table 1:** Comparison between the numerically calculated and the experimentally measured FWHMs in Fig. 4 (unit: mm).

	Numerically calculated FWHM	Experimentally measured FWHM	Rayleigh diffraction limit (The focusing limit)
Conventional FZP lens	1.24	1.37	1.28
Optimized SOAL	0.78	0.84	

### Transmission efficiency of the optimized SOAL

In order to accurately evaluate the performance of an acoustic lens, the ratio of acoustic power in main focusing area to the total incident acoustic power (i.e., transmission efficiency) should be considered, in addition to the FWHM corresponding to the spatial resolution^41^. Therefore, we defined the indicator of transmission efficiency (*TE*) as Eq. (6), and then compared the performance of acoustic lenses by using this indicator.

6 $$TE={f}_{AP}^{t}\times {f}_{AP}^{f}\times 100\,( \% )$$

7 $${\rm{where}},\,{f}_{AP}^{f}=\frac{{\iint }_{{S}_{M}}{|p|}^{2}/2{\rho }_{w}{c}_{w}dA}{{\iint }_{{S}_{E}}{|p|}^{2}/2{\rho }_{w}{c}_{w}dA}=\frac{{\iint }_{-FWHM/2}^{+FWHM/2}{|p|}^{2}/2{\rho }_{w}{c}_{w}dA}{{\iint }_{-3\,mm}^{+3\,mm}{|p|}^{2}/2{\rho }_{w}{c}_{w}dA}$$

In this equation, ${f}_{AP}^{t}$ is the fraction of acoustic power transmitted through the acoustic lens to the total incident acoustic power. Also ${f}_{AP}^{f}$ is the fraction of acoustic power in the main focusing area to the acoustic power transmitted through the acoustic lens. First, ${f}_{AP}^{t}$ can be determined by the ratio of acoustically transparent area to the entire area of acoustic lens with the assumption of no thermal-viscous loss. In the case of the conventional FZP lens, the area of acoustically transparent region occupies about 50% of the entire area. Intuitively, thus, it is natural to expect that approximately 50% of the total incident acoustic power is assuredly to be transmitted. In this case, ${f}_{AP}^{t}$ is 0.5. Next, ${f}_{AP}^{f}$ can be calculated as the ratio of the energy portions occupied by the main focusing area (*S*_*M*_) and the entire area (*S*_*E*_) in the acoustic intensity profile at the focal length (*F*), as expressed by Eq. (7). To calculate these areas from the acoustic intensity profile numerically, a trapezoidal integration scheme was utilized.

**Figure 5:**
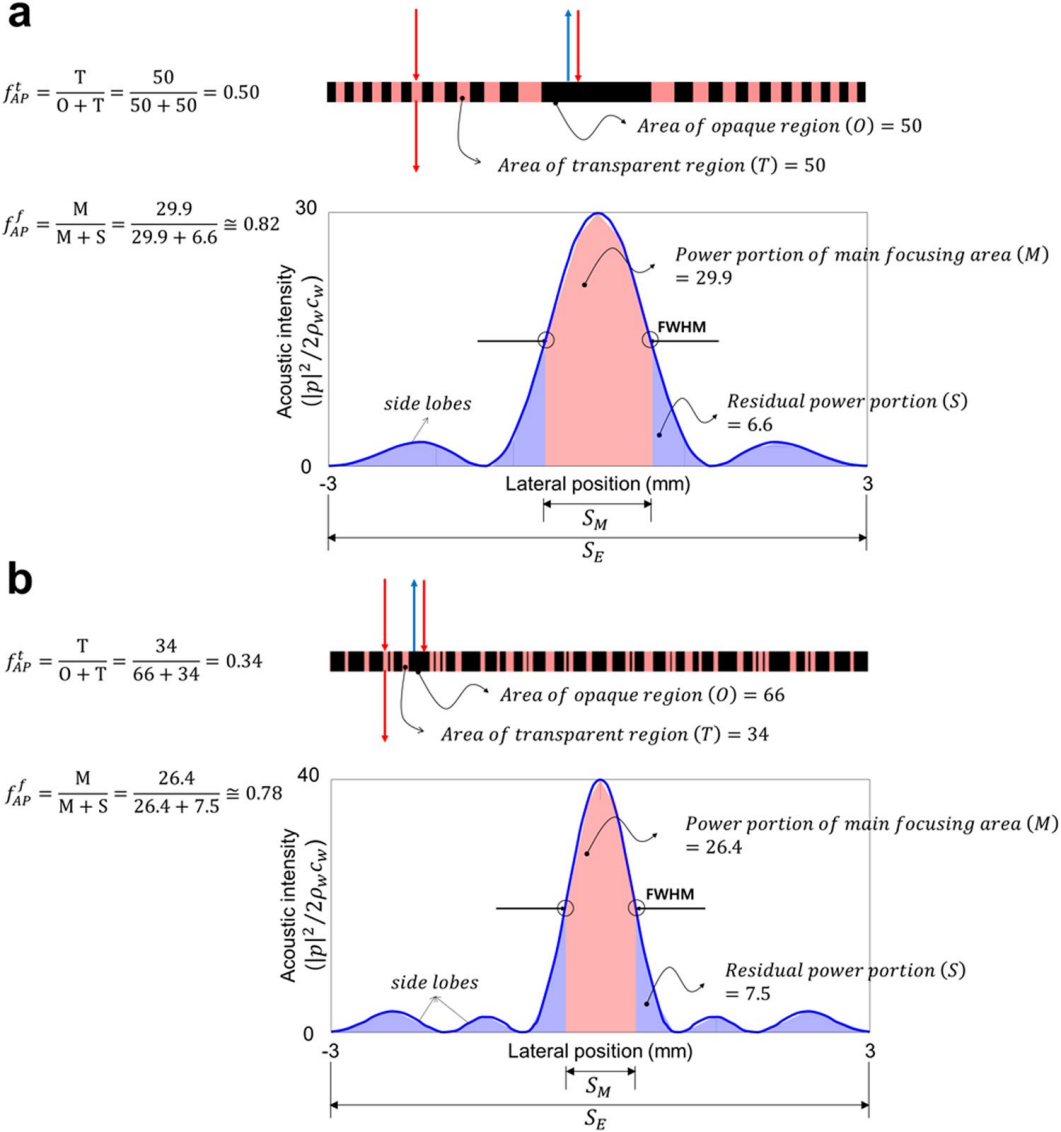
Calculation of transmission efficiency for both the conventional FZP lens and the optimized SOAL. **(a)** Conventional FZP lens **(b**) Optimized SOAL. In each (a,b), the upper panel represents the layout of acoustic lenses and the lower panel represents the acoustic intensity profile at the focal length (*F*) for the acoustic lenses.

We calculated *TE*s for both the conventional FZP lens and the optimized SOAL by using the scheme as mentioned above. For conventional FZP lens, ${f}_{AP}^{t}$ is 0.5 and ${f}_{AP}^{f}$ is about 0.82. Thus, the *TE* of the conventional FZP lens is about 41.0% (Fig. 5a). By the same approach, for the optimized SOAL with single layer, we obtain 0.34 of ${f}_{AP}^{t}$ and 0.78 for ${f}_{AP}^{f}$ (Fig. 5b). As a result, the *TE* of the optimized SOAL is about 26.5%. The results of this transmission efficiency test are summarized in Table 2. The optimized SOAL has much better performance than the conventional FZP lens in terms of FWHM. However, from the perspective of transmission efficiency, the conventional FZP lens is more efficient compared to the optimized SOAL.

**Table 2:** Comparison of transmission efficiency between the conventional FZP lens and the optimized SOAL.

	${{\boldsymbol{f}}}_{{\boldsymbol{AP}}}^{{\boldsymbol{t}}}$	${{\boldsymbol{f}}}_{{\boldsymbol{AP}}}^{{\boldsymbol{t}}}$	${{\boldsymbol{f}}}_{{\boldsymbol{AP}}}^{{\boldsymbol{t}}}{\boldsymbol{\times }}{{\boldsymbol{f}}}_{{\boldsymbol{AP}}}^{{\boldsymbol{t}}}{\boldsymbol{\times }}\mathrm{100}\,({\boldsymbol{ \% }})$
Conventional FZP lens	0.50	0.82	41
Optimized SOAL	0.34	0.78	26.5

From the transmission efficiency test, it is worth noting here that the larger side lobe is always accompanied to achieve an even narrower FWHM beyond the Rayleigh diffraction limit. Thus, the transmission efficiency of an acoustic lens with a narrower FWHM is generally more decreased. The physical basis for this phenomenon can be more reliably supported from^4^. Consequently, the focusing performance and the transmission efficiency have a trade-off relationship to each other. This means that it is necessary to select the acoustic lens which is best matched to the purpose of the system to be applied. In the future, in order to compensate for this degradation problem of transmission efficiency, the most physically feasible solution is to maximize the fraction of acoustic power transmitted through acoustic lens to the total incident acoustic power $({f}_{AP}^{t})$. To achieve this goal, an impedance-matched acoustic metasurface or resonance-based energy amplification could be applied.

Finally^4^ clarified more reasonable criteria about overcoming the diffraction limit in the focusing application as the following two: (1) The smallest period of the spatial Fourier components in the image plane is less than λ/2, (2) More than approximately 50% of the incident acoustic power is focused in the main focusing area. Indeed, our study has focused on the first criterion. The optimized SOAL shows that the acoustic intensity profile in the main focusing area in the image plane can locally oscillate faster than the highest spatial Fourier component of original acoustic wave. In other words, our optimized SOAL can overcome the diffraction limit under the first criterion. On the other hand, the optimized SOAL violates the second criterion, but this issue is not big for some imaging applications.

## Discussion

In this study, we optimized an ultrathin (0.14λ) SOAL with a subwavelength focusing feature that can focus incident acoustic power into a subwavelength area in water. To design the optimal layout of the SOAL systematically, we applied an inverse design methodology known as topology optimization. By utilizing the proposed inverse design methodology, the SOAL was designed to maximize the focusing performance of the acoustic power in terms of the FWHM. We confirmed for the first time from the experimental realization of the SOAL that the optimized SOAL can overcome the diffraction limit and achieve subwavelength focusing with the FWHM of ~0.40λ/NA ≃ 0.84 mm (for our SOAL, NA = 0.707). By adding constraints on the minimization of side lobes to the current topology optimization formulation, the SOAL exhibiting a much improved focusing limit and better resolution (e.g., FWHM of $\sim 0.20{\rm{\lambda }}/{\rm{NA}}$) can be achieved. The optimized SOAL in this study shows the transmission efficiency of 26.5%. For more practical diagnostic and therapeutic processes in clinical situations, the acoustic power should be focused at a position far (i.e., ${\rm{z}}\gg {\rm{\lambda }}$) from the acoustic lens. Specifically, the acoustic lens should have a long focal length. Since the optimized SOAL can focus acoustic power into a position approximately 16λ far away from that lens, we expect that it can offset some of the disadvantages of near-field imaging approaches, including those using several AMM-based lenses. Although a solution to the thermal-viscous loss effect owing to the very narrow micro-slits remains to be discovered, the optimized SOAL has a great advantage in terms of practical applications. Moreover, the optimized SOAL has several other benefits, such as the potential for low-power therapy, far-field focusing with a long focal length, and control of the focal length simply by replacing the optimized SOAL. Therefore, it will likely improve the therapeutic performance of High-Intensity Focused Ultrasound (HIFU)/High-Intensity Therapeutic Ultrasound (HITU)^42^. The optimized SOAL will also be applied to acoustic microscopy in order to realize super-resolution acoustic imaging in practice. The concept of the optimized SOAL can be extended to various wave-based systems (e.g., elastic, acoustic, and optical waves) through a design process similar to that proposed here.

## Methods

### Finite element numerical simulation

Throughout the paper, the Finite Element Method (FEM) based on the commercial software COMSOL Multiphysics and MATLAB is employed for both the numerical analysis and the design optimization of the SOAL. The materials used in the simulations are water and SUS 303. A time-harmonic analysis is conducted at 1 MHz operational frequency to compute the acoustic field through the optimized SOAL. Second-order Sommerfeld absorbing boundary conditions are set on the exterior boundaries of the simulation domain to eliminate boundary-reflected acoustic waves. The source transducer is approximated by the plane wave boundary condition. The proper mesh element size (*h*_*max*_) is selected as the 1/10 of the wavelength (*λ*/10) through the convergence test. The convergence test result is shown in the Supplementary Note.

### Experimental setup

The experimental setup consists of a transducer, a hydrophone, an arbitrary waveform generator, an amplifier, and a water tank (Fig. 3c). The transducer (KRISS1MHz4p5, *Korea Research Institute of Standards and Science*) is used to create the 1 MHz frequency plane wave. A needle-type hydrophone with a diameter of 500 μm (*Precision Acoustics*) with a submersible preamplifier is used to measure the acoustic field accurately through an acoustic lens. We also use an arbitrary waveform generator (33250A, *Agilent Technologies*) to generate a 15-cycle tone-burst signal of 200 mV_*rms*_ at 1 MHz. To amplify the generated tone-burst signal, a RF amplifier (2100 L, *Electronics & Innovation, Ltd*.) is used. The transducer and needle-type hydrophone are installed in a tank with deionized and degassed water, and the arbitrary waveform generator and RF amplifier are installed outside the tank. The acoustic field through the acoustic lens is then measured by an acoustic intensity measurement system (AIMS III with Soniq software, *ONDA*).

## Electronic supplementary material

Supplementary Information
