# Supplementary material for: Realization of an ultrathin acoustic lens for subwavelength focusing in the megasonic range
Source: Sci Rep. 2018 Jun 14;8:9131. doi: 10.1038/s41598-018-27312-5 (PMC6002413; doi:10.1038/s41598-018-27312-5)
Supplement: Supplementary file 1 — Supplementary Information [file 41598_2018_27312_MOESM1_ESM.docx]

**Supplementary Note for**

**“Realization of an ultrathin acoustic lens for subwavelength focusing in the megasonic range”**

**Jaeyub Hyun^1^, Yong Tae Kim^1*^, Il Doh^1^, Bongyoung Ahn^1^,**

**Kyungmin Baik^1^ and Sehwa Kim^2^**

^1^Center for Medical Convergence Metrology, Korea Research Institute of Standards and Science (KRISS), 267 Gajeong-ro, Yuseong-gu, Daejeon 34113, Republic of Korea

^2^Center for Nano-Bio Measurement, Korea Research Institute of Standards and Science (KRISS), 267 Gajeong-ro, Yuseong-gu, Daejeon 34113, Republic of Korea

*Corresponding author

Postal Address: Center for Medical Convergence Metrology, Korea Research Institute of Standards and Science (KRISS), 267 Gajeong-ro, Yuseong-gu, Daejeon 34113, Republic of Korea. E-mail Address: [ytkim@kriss.re.kr](mailto:ytkim@kriss.re.kr)

In this Supplementary Note, S1) a summary of many previous studies on planar acoustic lens is provided in a table form for easier readability. S2) In addition, through specific design examples, we present how the systematic design approach proposed in our manuscript can be effectively applied to a multilayer-type SOAL as well as the single-layer type of SOAL. S3) Also, the detailed dimension parameters of both a conventional FZP lens and the optimized single-layer type of SOAL are given. S4) The comparison between the finite element numerical simulation of the optimized SOAL with and without the shear deformation effect of SUS 303 is given. Through this comparison result, the reason why this study ignored the shear deformation effect is confirmed. S5) Next, the proposed systematic design approach is used for designing the SOAL with even narrower FWHM than ~0.4λ/NA. We show from these results that in principle the SOAL can achieve any value of FWHM at the expense of side lobes. S6) Finally, we carry out a convergence test to present the accuracy of finite element numerical simulation according to the maximum mesh element size.

**S1. Previous studies related to both the design and realization of a planar acoustic lens**

Many previous studies on the design and experimental realization of planar acoustic lenses, including SOAL types similar to that covered in our manuscript, are reviewed. Their results are summarized in Table S1. It is important to note, as mentioned in the manuscript, that the Numerical Aperture (NA) should be considered when accurately quantifying the focusing performance of the acoustic lens. Therefore, the data in the last column in Table S1 represents the most meaningful indicator of the focusing performance. Through this review, we have confirmed that the optimized SOAL can be a suitable solution for subwavelength focusing in the megasonic range (> 1 MHz), and its realization can make a significant contribution to the fields of ultrasound focusing and imaging.

**Table S1. Review of previous studies of planar acoustic lenses**

**In this table, only experimentally realized acoustic lenses are presented.*

**NA is determined by both the radius of source transducer (*$r_{source}$*) and the target focal length* (*F*) as *N*$A= \sin\left( {tan}^{-1}\left( {r_{source}}/F \right) \right)$.

| Reference | Operational medium | Operational frequency (kHz) | Lens thickness  (mm) | Radius of source transducer (mm) | Target focal length (mm) | Achieved lateral FWHM  (mm) | Achieved lateral FWHM including NA  (mm) |
| --- | --- | --- | --- | --- | --- | --- | --- |
| D.W. Schindel (1997) [1] | air | 580 | 0.125 | 8.14 | 7.5 | 1.36λ | 1.00λ/NA |
| T.E.G. Alvarez-Arenas (2016) [2] | air | 400 | 0.1 | 15 | 33.5 | 2.91λ | 1.19λ/NA |
| J.T. Welter (2011) [3] | air | 85 | 3 | 20 | 2.5 | 0.88λ | 0.87λ/NA |
| M. Moleron (2014) [4] | air | 5.85 | 25 | 210 | 71 | 0.61λ | 0.58λ/NA |
| B. Yuan (2015) [5] | air | 2 | 16 | 165 | 100 | 0.70λ | 0.60λ/NA |
| Y. Li  (2014) [6] | air | 0.82 | 47 | 675 | 1,400 | 1.20λ | 0.52λ/NA |
| M.Z. Sleva (1994) [7] | water | 5,000 | 0.111 | 17.3 | 52 | 3.02λ | 0.95λ/NA |
| H. Wang (2008) [8] | water | 2,000 | 0.5 | 5.68 | 10 | 1.26λ | 0.62λ/NA |
| C. Rubio (2017) [9] | water | 200 | 2 | 87.7 | 104 | 1.24λ | 0.80λ/NA |
| D.C. Calvo (2015) [10] | water | 200 | 3 | 16.6 | 8.75 | 0.68λ | 0.61λ/NA |

**S2. Application of the proposed systematic design methodology to a multilayer-type SOAL**

In addition to the optimization of SOAL with a single layer, we optimized a multilayer-type SOAL according to the number of layers by employing the proposed systematic design methodology (Fig. S1a). The spacing between each layer of the SOAL is 0.2 mm. The result in Fig. S1b indicates that the focusing performance of the SOAL is enhanced as the number of layers is increased. In other words, the multilayer-type SOAL constructs an ultrasonic super-oscillatory needle by lengthening the axial FWHM and narrowing the lateral FWHM of the focused ultrasound, similar to an optical needle [11]. The ultrasonic super-oscillatory needle has the advantage of robust imaging along the axial direction. Moreover, the multilayer-type SOAL has a valuable advantage with regard to fabrication. To improve the focusing performance of the single-layer SOAL even more, more rings are needed. The layout of the single-layer SOAL should also be precisely designed [12]. However, as the number of rings is increased, it becomes difficult to realize a single-layer SOAL through an existing fabrication method such as laser-cutting and/or photo-etching. Hence, the multilayer-type SOAL can be a feasible means of resolving these fabrication challenges.


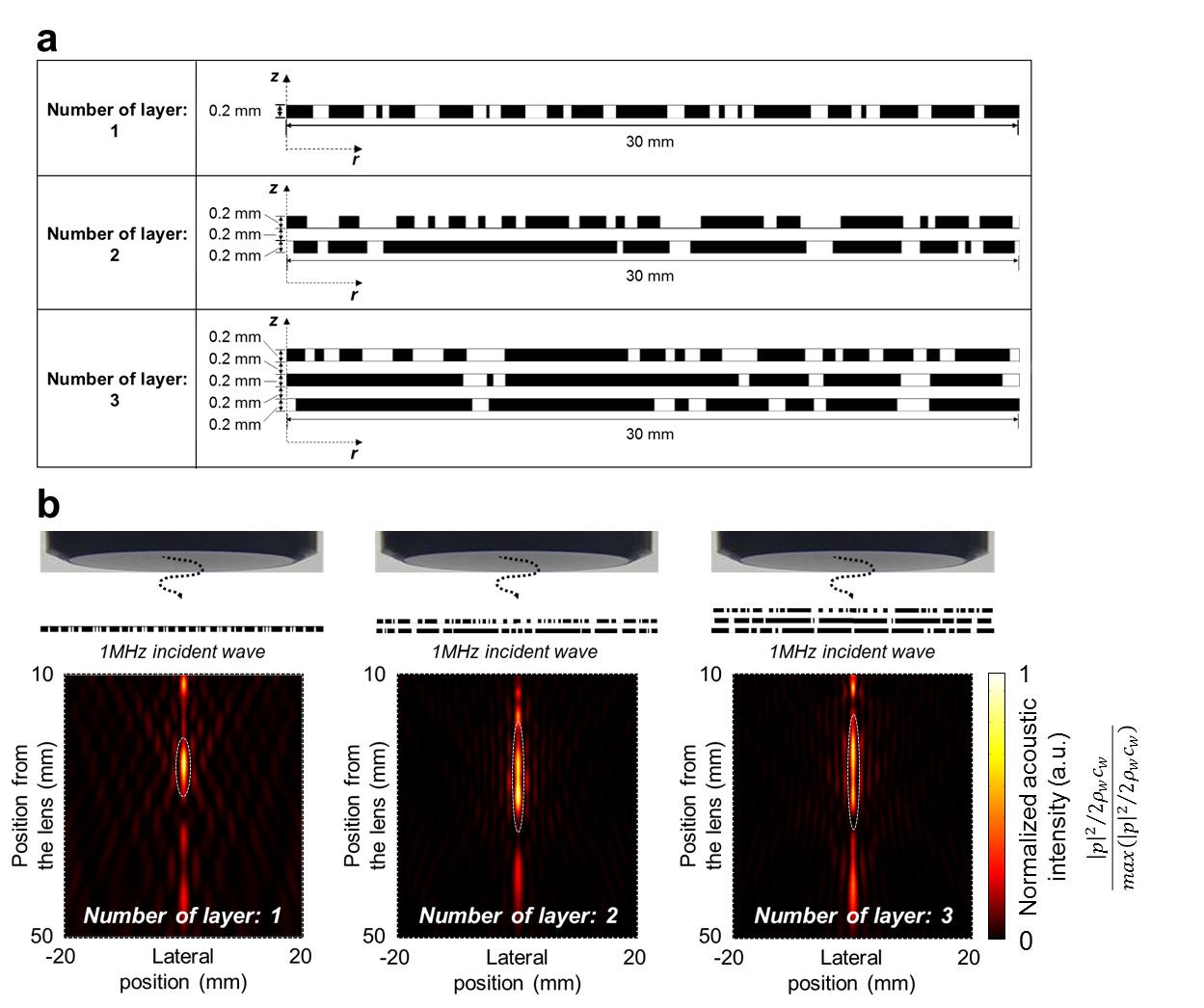


**Figure S1. Comparison of the optimal layouts of the binary ring mask-type SOAL according to the number of layers: (a)** Configuration of the optimal layout of the optimized SOAL utilizing the proposed systematic design approach. **(b)** Acoustic intensity normalized by the maximum value of intensity for each optimized SOAL. Left panel: optimized SOAL with a single layer. Middle panel: optimized SOAL with a double layer. Right panel: optimized SOAL with a triple layer.

**S3. The detailed dimensions of both the conventional FZP lens and the optimized single-layer SOAL**

Table S2 below shows the detailed dimensions of both the conventional FZP lens and the optimized single-layer SOAL described in this study. The dimensions of the conventional FZP lens are obtained from an analytical equation (Eq. (4)) presented in the manuscript. The configurations of both lenses are shown in Fig. S2.


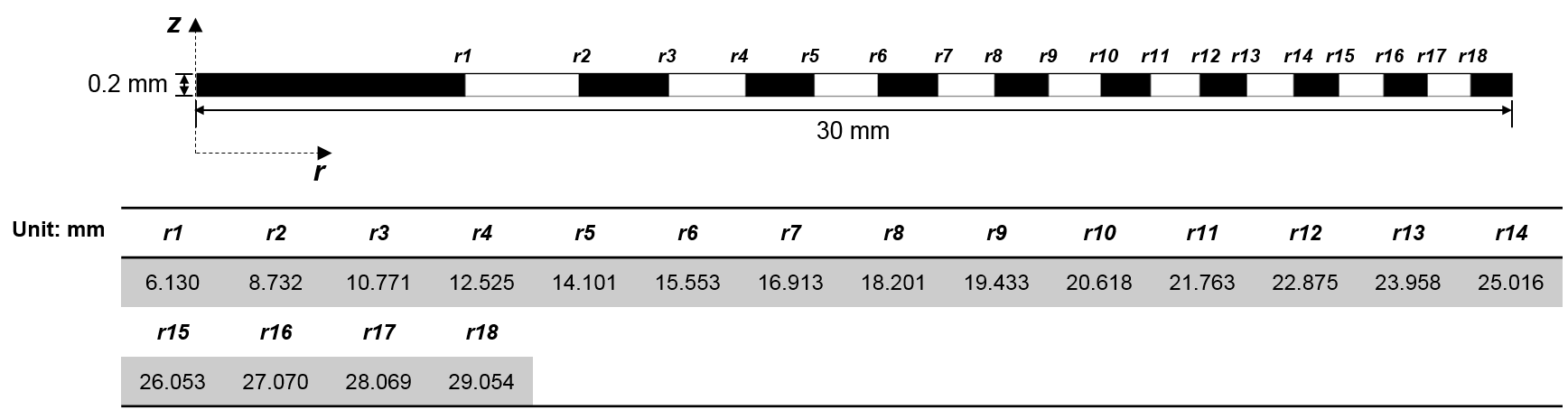

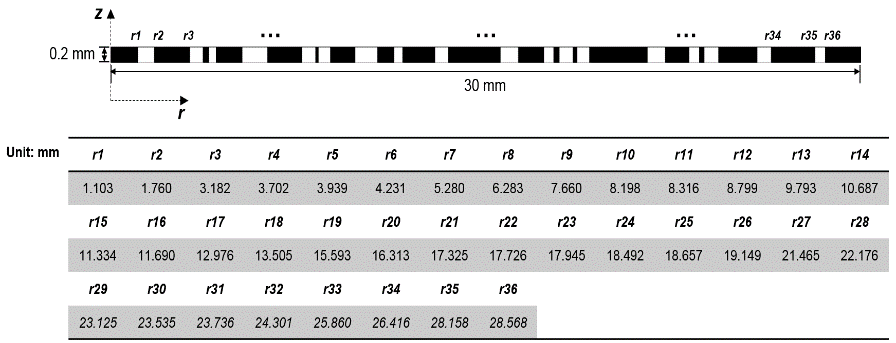


**Figure S2**. **Configurations of the conventional FZP lens (upper panel) and the optimized single-layer SOAL (lower panel)**

**Table S2. The detailed dimensions of both the conventional FZP lens and the optimized single-layer SOAL. The first two columns are for the conventional FZP lens and the next two columns are for the optimized SOAL (unit: mm).**

| **Conventional FZP lens (mm)** | | **Optimized singles layer-type SOAL (mm)** | |
| --- | --- | --- | --- |
| **r1** | 6.13 | **r1** | 1.10 |
| **r2** | 8.74 | **r2** | 1.76 |
| **r3** | 10.78 | **r3** | 3.18 |
| **r4** | 12.53 | **r4** | 3.70 |
| **r5** | 14.11 | **r5** | 3.94 |
| **r6** | 15.56 | **r6** | 4.23 |
| **r7** | 16.92 | **r7** | 5.28 |
| **r8** | 18.21 | **r8** | 6.28 |
| **r9** | 19.44 | **r9** | 7.66 |
| **r10** | 20.63 | **r10** | 8.20 |
| **r11** | 21.77 | **r11** | 8.32 |
| **r12** | 22.88 | **r12** | 8.80 |
| **r13** | 23.97 | **r13** | 9.79 |
| **r14** | 25.03 | **r14** | 10.69 |
| **r15** | 26.06 | **r15** | 11.33 |
| **r16** | 27.08 | **r16** | 11.69 |
| **r17** | 28.08 | **r17** | 12.98 |
| **r18** | 29.07 | **r18** | 13.51 |
|  | | **r19** | 15.59 |
|  |  | **r20** | 16.31 |
|  |  | **r21** | 17.33 |
|  |  | **r22** | 17.73 |
|  |  | **r23** | 17.95 |
|  |  | **r24** | 18.49 |
|  |  | **r25** | 18.66 |
|  |  | **r26** | 19.15 |
|  |  | **r27** | 21.47 |
|  |  | **r28** | 22.18 |
|  |  | **r29** | 23.13 |
|  |  | **r30** | 23.54 |
|  |  | **r31** | 23.74 |
|  |  | **r32** | 24.30 |
|  |  | **r33** | 25.86 |
|  |  | **r34** | 26.42 |
|  |  | **r35** | 28.16 |
|  |  | **r36** | 28.57 |

**S4. Comparison between the numerical simulation of the optimized SOAL with and without the shear deformation effect**

In this study, the shear deformation effect of elastic solid (i.e., SUS 303) was not considered during numerical simulation. First of all, this is because SUS 303 can be regarded as acoustically rigid due to its large impedance contrast compared to the surrounding medium (i.e., water) [13]. Moreover, even if the shear deformation appears in SUS 303, the shear component of wave is hardly transferred to the water supporting only the longitudinal component of that [14]. Furthermore, there is no mode-conversion from longitudinal to shear waves for acoustic wave incident normal to the acoustic lens [15]. Therefore, the shear deformation effect in SUS 303 can be ignored during the numerical simulation and optimization process of the acoustic lens. However if the impedance of a material used to model an acoustically opaque region is comparable to that of the surrounding medium (i.e., water), or if the real-world phenomena such as structural resonances must be considered, the coupling effect by this shear deformation should be included in numerical simulation. For this purpose, Acoustic-Structure Interaction (ASI) modeling with shear deformation can be used.

In order to clarify the reason for ignoring the shear deformation effect, we compared the numerically calculated acoustic intensity fields between the optimized SOAL based on pure acoustic modeling without shear deformation and that based on ASI modeling with shear deformation. The mechanical properties of SUS 303 used for ASI modeling are as follows: Young’s modulus (E) = 193 GPa, Mass density (ρ) = 8,000 kg/m^3^, and Poisson’s ratio (ν) = 0.25 [16] . As shown in the numerically calculated acoustic intensity fields in Figs. S3a and S3b these two models show almost the same results. Meanwhile, the maximum value of the acoustic intensity of SOAL based on ASI modeling (Fig. S3d) is somewhat smaller than that of SOAL based on pure acoustic modeling (Fig. S3c). This is because the structure of SOAL is partially excited by the incident acoustic pressure. However, this small discrepancy in acoustic intensity value has little effect on the designs of acoustic lenses (e.g., SOAL), since the important factor is the overall distributed shape of the acoustic field.


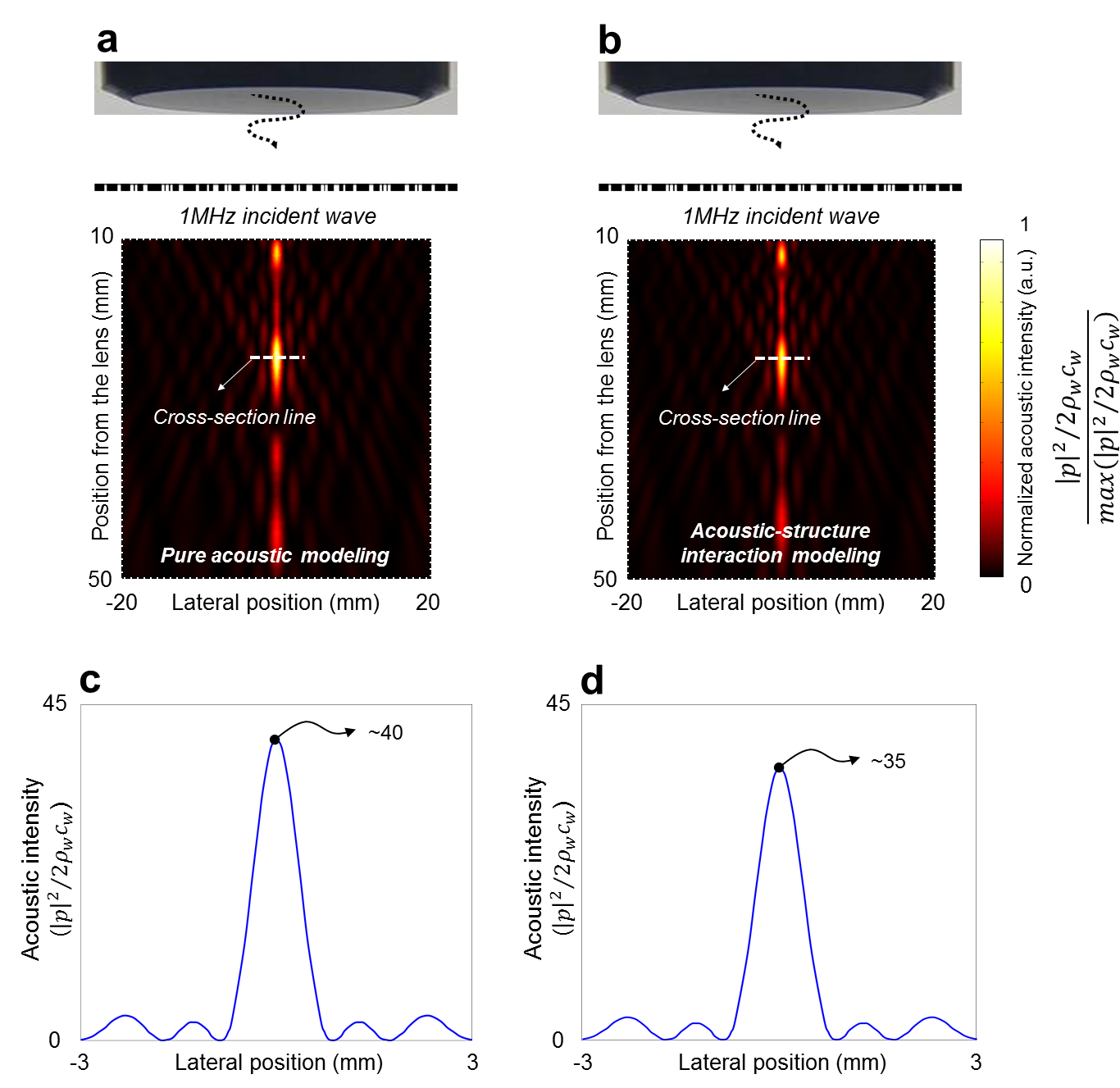


**Figure S3. Numerically calculated 2D acoustic intensity fields and cross-sectional plot for the optimized SOAL with and without the shear deformation effect: (a, c)** Optimized SOAL based on pure acoustic modeling without shear deformation **(b, d)** Optimized SOAL based on ASI modeling with shear deformation.

**S5. Examination of the design possibility of SOAL with even narrower FWHM**

The inverse design method proposed in this study can be utilized to achieve the even narrower FWHM (e.g.,$\sim0.20\lambda/{NA}$) than$\sim0.40\lambda/{NA}$. However, when the narrower FWHM is obtained, the larger side lobe is likely to appear. To illustrate this issue clearly, the inverse design of the SOAL was performed by setting the target FWHM (${FWHM}_{tar.}$) to$0.28\lambda/{NA}$,$0.24\lambda/{NA}$,$0.20\lambda/{NA}$ and $0.12\lambda/{NA}$, in addition to the SOAL with the FWHM of $\sim0.40\lambda/{NA}$ covered in this manuscript. The side lobe is increased sharply as the target FWHM of main focusing area is decreased, as shown in Fig. S4. Particularly, in the case of the SOAL with the FWHM of $0.12\lambda/{NA}$ in Fig. S4d, the acoustic intensity in the side lobe is much larger than that in the main lobe. This large side lobe effect is inevitable in designing the super-oscillatory optical/acoustic lenses with very narrow FWHM [17]. Despite the possibility of designing the SOAL with narrower FWHM, the reason that this study does not focusing on the design of SOAL with FWHM narrower than $\sim0.40\lambda/{NA}$ comes from the following three perspectives: *1) imaging (diagnosis), 2) therapeutic, and 3) thermal-viscous loss.* A detailed description of these has been explained below.


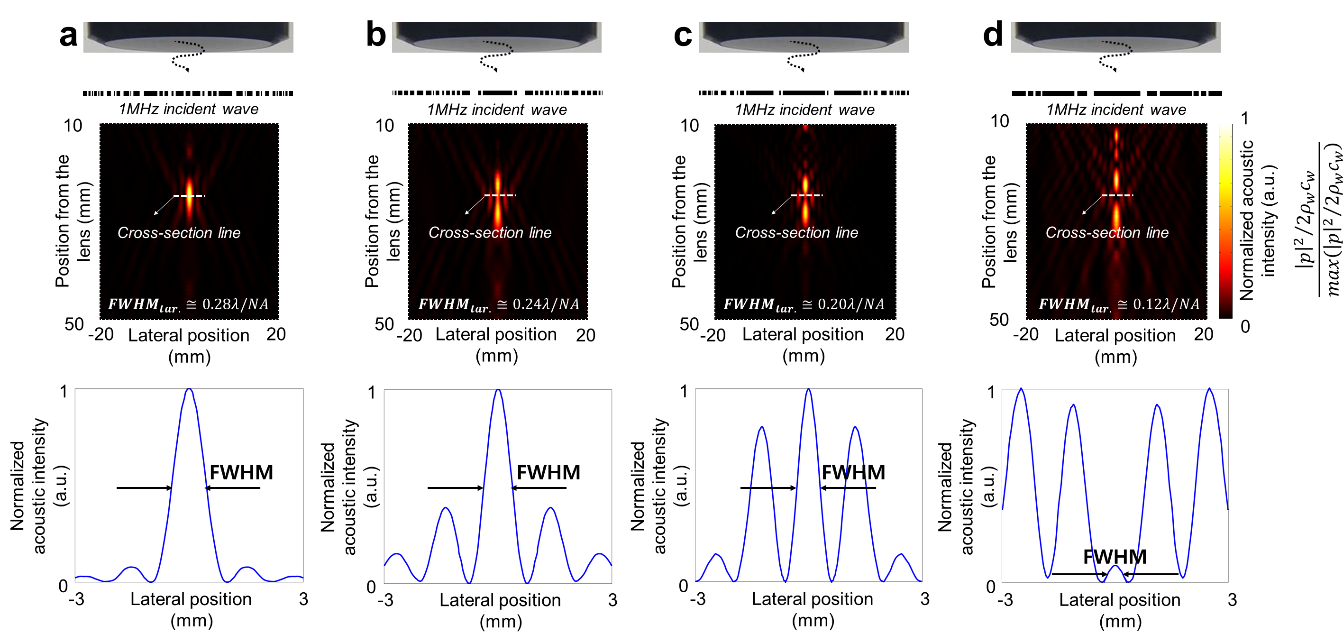


**Figure S4. The inverse design results of the SOALs with the target FWHMs (**$\boldsymbol{FWHM}_{\boldsymbol{tar.}}$**) narrower than**$\boldsymbol{\sim0.40}\boldsymbol{\lambda}/\boldsymbol{NA}$**: (a)**${FWHM}_{tar.}=0.28\lambda/{NA}$, **(b)**${FWHM}_{tar.}=0.24\lambda/{NA}$, **(c)**${FWHM}_{tar.}=0.20\lambda/{NA}$, **(d)**${FWHM}_{tar.}=0.12\lambda/{NA}$. Here, the upper panel represents the acoustic intensity fields and the lower panel represents the cross-sectional plot of the acoustic intensity fields (i.e., acoustic intensity profile) near the main focusing area. Please note that as the target FWHM become narrow, the acoustic intensity in the side lobe gradually is increased.

***1) In terms of imaging (diagnosis)***

For some imaging applications, the false image caused by side lobes can be effectively eliminated. For instance, in optical imaging, light scattered by a side lobe can be suppressed using a confocal imaging technique. The confocal imaging technique is able to reject out-of-focusing light using a special selection mechanism based on the pinhole aperture. Therefore, the confocal laser scanning microscopy using this technique is characterized by the rejection mechanism, referred to as optical sectioning, which only shows clearly the imaging objects presented in the main focusing area [18]. Hence, this makes it possible to produce the super-resolution imaging using only the super-focusing feature of the main focusing area. In this regard, the confocal laser scanning microscopy is widely used in the field of optical imaging. Indeed, if the very small amount of energy (i.e., very few photons) in the main focusing area accompanied with a large side lobe is available for imaging, there is no resolution limit theoretically [19, 20]. Of course, there are practical limitations due to a number of uncertain factors such as the error of the optical system including a lens. Based on our knowledge, there is no commercially available acoustic microscopes using this confocal imaging technique. Therefore, this study restricts the main target application systems of the SOAL to general ultrasonic transducers that are widely used in clinical (e.g., abdominal imaging) and industrial (e.g., nondestructive evaluation) fields. In conventional acoustic imaging method based on the pulse-echo scheme, if the acoustic waves from the side and main lobes are mixed in the echo signal, some false images can be formed. Eventually, suppression of side lobe becomes very important issue.

***2) In terms of therapeutic***

In the therapeutic systems such as High-Intensity Focused Ultrasound (HIFU) where acoustic energy is focusing on the lesion and instantaneous thermal/mechanical effects are used to non-invasively treat that, acoustic excitation on areas other than the target lesion by side lobes could cause side effects. Thus, it is important to not only precisely control the main focusing area but also suppress the side lobe, for designing the acoustic lens for HIFU application.

***3) In terms of thermal-viscous loss***

If a narrower slit is designed to achieve a narrower FWHM, the thermal-viscous loss will be significant, resulting in the degradation of focusing performance. Therefore, there is a need to design the SOAL for minimizing this thermal-viscous loss effect from the perspective of practical fabrication and application.

Bring it all together, this study limited the target FWHM to$\sim0.40\lambda/{NA}$ in order to design the SOAL exhibiting small side lobe and thermal-viscous loss effect.

**S6. Accuracy of numerical simulation according to the maximum mesh element size**

We performed the convergence test of finite element numerical simulation according to the maximum mesh element size ($h_{max}$). The list of maximum mesh element sizes considered for the numerical simulation is as follows:$2\lambda$, $\lambda$, $\lambda/2$, $\cdots$, $\lambda/{14}$. First, as$h_{max}$ is decreased, the shape of acoustic intensity fields calculated from the numerical models is gradually smoother (Upper panel in Fig. S5). Next, we plot the variation trend of the maximum acoustic intensity value in the target main focusing area according to $h_{max}$ (Lower panel in Fig. S5), in order to quantify the convergence of the numerical solution. As shown in Fig. S5, the numerical models based on $h_{max}$ smaller than $\lambda/6$ show that the maximum acoustic intensity values are almost the same one another. In fact, in the field of numerical acoustics, a usual rule of thumb is that six (linear) finite elements per wavelength ($\lambda/6)$ are sufficient in order to obtain accurate results [21]. This can be also confirmed through our convergence test. In this study, we tried to perform a more accurate finite element numerical simulation by using$h_{max}$ of $\lambda/{10}$ smaller than$\lambda/6$.


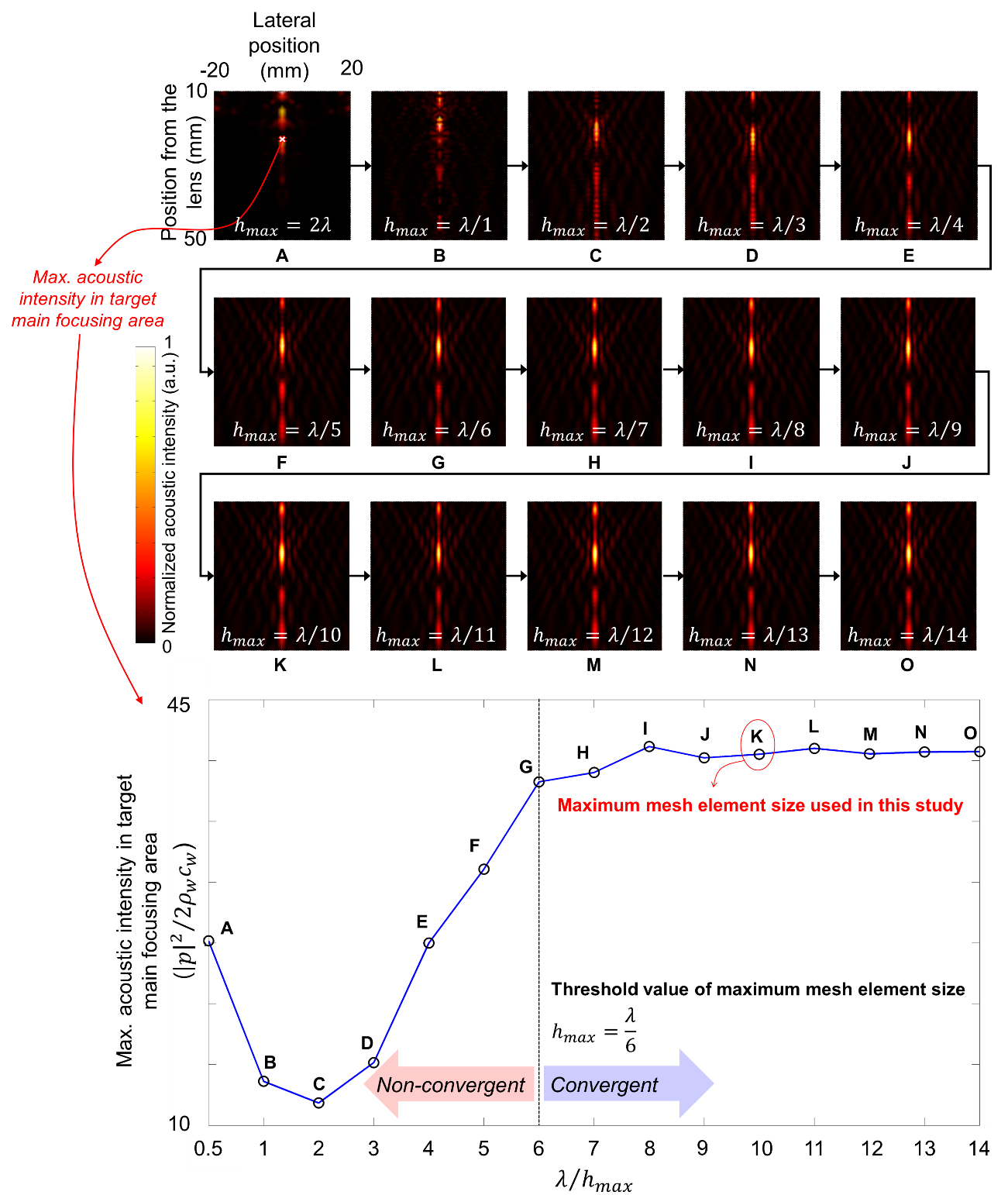


**Figure S5. Convergence test of finite element numerical simulation according to the maximum mesh element size (**$\boldsymbol{h}_{\boldsymbol{max}}\boldsymbol{)}$**.** Here, A to O in the upper panel are the acoustic intensity fields calculated from the numerical models corresponding to points from A to O in the convergence plot in the lower panel.

**References**

| 1. | Schindel, D. W., Bashford, A. G. & Hutchins, D. A. Focussing of ultrasonic waves in air using a micromachined Fresnel zone-plate. *Ultrasonics,* **35**, 275-285 (1997). |
| --- | --- |
| 2. | Alvarez-Arenas, T. E. G., Camacho, J. & Fritsch, C. Passive focusing techniques for piezoelectric air-coupled ultrasonic transducers. *Ultrasonics,* **67**, 85-93 (2016). |
| 3. | Welter, J. T. *et al.* Focusing of longitudinal ultrasonic waves in air with an aperiodic flat lens. *J. Acoust. Soc. Am.,* **130**, 2789-2796 (2011). |
| 4. | Moleron, M., Garcia, M. S. & Daraio, C. Acoustic Fresnel lenses with extraordinary transmission. *Appl. Phys. Lett.,* **105**, 114109 (2014). |
| 5. | Yuan, B., Cheng, Y. & Liu, X. Conversion of sound radiation pattern via gradient acoustic metasurface with space-coiling structure. *Appl. Phys. Express,* **8**, 027301 (2015). |
| 6. | Li, Y. *et al.* Three-dimensional ultrathin planar lenses by acoustic metamaterials. *Sci. Rep.,* **4**, 6830 (2014). |
| 7. | Sleva, M. Z., Hunt, W. D. & Briggs, R. D. Focusing performance of epoxy- and air-backed polyvinylidene fluoride Fresnel zone plates. *J. Acoust. Soc. Am.,* **96**, 1627-1633 (1994). |
| 8. | Wang, H., Xing, D. & Xiang, L. Photoacoustic imaging using an ultrasonic Fresnel zone plate transducer. *J. Phys. D: Appl. Phys.,* **41**, 095111 (2008). |
| 9. | Rubio, C. *et al.* Pinhole zone plate lens for ultrasound focusing. *Sensors,* **17**, 1690 (2017). |
| 10. | Calvo, D. C., Thangawng, A. L., Nicholas, M. & Layman, C. N. Thin Fresnel zone plate lenses for focusing underwater sound. *Applied Physics Letters,* **107**, 014103 (2015). |
| 11. | Rogers, E. T. F. *et al.* Super-oscillatory optical needle. *Appl. Phys. Lett.,* **102**, 031108 (2013). |
| 12. | Yuan, G. *et al.* Planar super-oscillatory lens for sub-diffraction optical needles at violet wavelengths. *Sci. Rep.,* **4**, 6333 (2014). |
| 13. | Duhring, M. B., Jensen, J. S. & Sigmund, O. Acoustic design by topology optimization. *J. Sound Vib.,* **317**, 557-575 (2008). |
| 14. | Noguchi, Y. *et al.* An acoustic metasurface design for wave motion conversion of longitudinal waves to transverse waves using topology optimization. *Appl. Phys. Lett.,* **107**, 221909 (2015). |
| 15. | Kohler, W., Papanicolaou, G. & White, B. Localization and mode conversion for elastic waves in randomly layered media I. *Wave Motion,* **23**, 1-22 (1996). |
| 16. | "303 Stainless Steel Mechanical Properties," E-Z LOK, 2018. https://www.ezlok.com/303-stainless-steel-properties. |
| 17. | Rogers, E. T. F. & Zheludev, N. I. Optical super-oscillations: sub-wavelength light focusing and super-resolution imaging. *J. Opt.,* **15**, 094008 (2013). |
| 18. | Hutzler, P. Tissue localization of phenolic compounds in plants by confocal laser. *J. Exp. Bot.,* **49**, 953-965 (1998). |
| 19. | Maznev, A. A. & Wright, O. B. Upholding limit in the focusing of light and sound. *Wave Motion,* **68**, 182-189 (2017). |
| 20. | Tang, D. E. A. Ultrabroadband superoscillatory lens coposed by plasmonic metasurfaces for subdiffraction light focusing. *Laser Photonics Rev.,* **9**, 713-719 (2015). |
| 21. | Marburg, S. Six boundary elements per wavelength: Is that enough?. *J. Comput. Acoust.,* **10**, 25-51 (2002). |
